# Tapping the biosynthetic potential of marine *Bacillus licheniformis* LHG166, a prolific sulphated exopolysaccharide producer: structural insights, bio-prospecting its antioxidant, antifungal, antibacterial and anti-biofilm potency as a novel anti-infective lead

**DOI:** 10.3389/fmicb.2024.1385493

**Published:** 2024-04-10

**Authors:** Nada K. Alharbi, Zahraa Falah Azeez, Haitham Mohammed Alhussain, Aisha M. A. Shahlol, Mona Othman I. Albureikan, Mohamed Gamal Elsehrawy, Ghfren S. Aloraini, Mohammad El-Nablaway, Elham Mohammed Khatrawi, Ahmed Ghareeb

**Affiliations:** ^1^Department of Biology, College of Science, Princess Nourah Bint Abdulrahman University, Riyadh, Saudi Arabia; ^2^Collage of Biotechnology, University of Al-Qadisiyah, Diwaniyah, Iraq; ^3^Department of Public Health and Infection Control, King Fahad Hospital, Alhofuf, Saudi Arabia; ^4^Department of Medical Laboratory Technology, Faculty of Medical Technology, Wadi-Al-Shatii University, Brack, Libya; ^5^Department of Biological Sciences, Faculty of Science, King Abdulaziz University, Jeddah, Saudi Arabia; ^6^College of Nursing, Prince Sattam Bin Abdelaziz University, Al-Kharj, Saudi Arabia; ^7^Faculty of Nursing, Port Said University, Port Said, Egypt; ^8^Department of Medical Laboratory Sciences, College of Applied Medical Sciences, Prince Sattam bin Abdulaziz University, Al-Kharj, Saudi Arabia; ^9^Department of Medical Biochemistry, Faculty of Medicine, Mansoura University, Mansoura, Egypt; ^10^Department of Basic Medical Sciences, College of Medicine, AlMaarefa University, Riyadh, Saudi Arabia; ^11^Department of Basic Medical Sciences, College of Medicine, Taibah University, Madinah, Saudi Arabia; ^12^Botany and Microbiology Department, Faculty of Science, Suez Canal University, Ismailia, Egypt

**Keywords:** microbial metabolites, natural products, drug discovery, antimicrobial resistance, anticandidiasis, antifungal, anti-biofilm

## Abstract

The escalating global threat of antimicrobial resistance necessitates prospecting uncharted microbial biodiversity for novel therapeutic leads. This study mines the promising chemical richness of *Bacillus licheniformis* LHG166, a prolific exopolysaccharide (EPSR2-7.22 g/L). It comprised 5 different monosaccharides with 48.11% uronic acid, 17.40% sulfate groups, and 6.09% N-acetyl glucosamine residues. EPSR2 displayed potent antioxidant activity in DPPH and ABTS^+^, TAC and FRAP assays. Of all the fungi tested, the yeast *Candida albicans* displayed the highest susceptibility and antibiofilm inhibition. The fungi *Aspergillus niger* and *Penicillium glabrum* showed moderate EPSR2 susceptibility. In contrast, the fungi *Mucor circinelloides* and *Trichoderma harzianum* were resistant. Among G+ve tested bacteria, *Enterococcus faecalis* was the most susceptible, while *Salmonella typhi* was the most sensitive to G−ve pathogens. Encouragingly, EPSR2 predominantly demonstrated bactericidal effects against both bacterial classes based on MBC/MIC of either 1 or 2 superior Gentamicin. At 75% of MBC, EPSR2 displayed the highest anti-biofilm activity of 88.30% against *B. subtilis*, while for G−ve antibiofilm inhibition, At 75% of MBC, EPSR2 displayed the highest anti-biofilm activity of 96.63% against *Escherichia coli*, Even at the lowest dose of 25% MBC, EPSR2 reduced biofilm formation by 84.13% in *E. coli*, 61.46% in *B. subtilis*. The microbial metabolite EPSR2 from *Bacillus licheniformis* LHG166 shows promise as an eco-friendly natural antibiotic alternative for treating infections and oxidative stress.

## 1 Introduction

Marine ecosystems constitute a substantial and distinctive habitat, including approximately 71% of the surface of the Earth’s planet (Costello and Chaudhary, 2017). Diverse and complex bacterial communities perform crucial functions necessary to maintain Earth’s ecosystem and stabilize the Earth’s biosphere (Sanchez et al., 2023). The synthesis of exopolysaccharide (EPS) by marine bacteria is a significant process that accounts for approximately 50% of the primary biosynthesis of organic compounds (García et al., 2022).

These EPSs play a crucial role in preserving marine ecosystems by facilitating several processes, such as the cycling of dissolved metals and organic carbon sequestration (Casillo et al., 2018). EPSs significantly promote organisms’ growth and survival in challenging ecological conditions. Also, EPSs play a crucial role in facilitating nutrient absorption, aggregation, adhesion to surfaces, and the formation of biofilms (Costa et al., 2018). Additionally, EPSs present microenvironments that shield bacteria from harsh environments, promote bacterial colonization and pathogenicity and ease the flow of genes and metabolites among bacterial communities (Arayes et al., 2023).

EPS is an externally formed organic macromolecule that resembles jelly or slime and is synthesized as a secondary metabolite. EPS can be synthesized as either a homo- or heteropolysaccharide polymer, with different subunit configurations depending on the species (Qamar et al., 2022). Most EPSs exhibit predominantly linear structures, characterized by their high molecular weights ranging from 1–3 ×10^5^ Da. Most known EPS is polyanionic, primarily because of the inclusion of inorganic groups (SO_4_^2−^ or PO_4_^3−^) and metal-linked pyruvate and uronic acid (Dogsa et al., 2005).

Microorganisms possess significant biosynthetic capabilities to synthesize a wide range of bioactive compounds characterized by distinct chemical architectures and functional characteristics. These compounds exhibited promising therapeutic potential. For instance, they inhibit the proliferation of pathogenic bacteria and fungi (Selim et al., 2022), impede the proliferation of malignant cells (Abdel-Wahab et al., 2022), scavenge reactive oxygen species (Alshawwa et al., 2022), diminish inflammation (Alharbi et al., 2023), and expedite the process of wound repair (Zaghloul and Ibrahim, 2022). These microbial bioactive metabolites encompass a diverse range of chemical structures, including peptides, lipopeptides, polypeptides, lactones, fatty acids, polyketides, isocoumarins, terpenoids, and exopolysaccharides (Zhou et al., 2019).

The antioxidative capacities of microbial exopolysaccharides have been studied and found to be substantial. The subunits of monosaccharides are classified as reducing sugars due to their possession of aldoses and ketoses or their ability to undergo interconversion between these two forms (Andrew and Jayaraman, 2020). For instance, EPSF6, isolated from *Bacillus Velezensis* AG6, was evaluated for antioxidant potential by DPPH, H_2_O_2_, and ABTS^+^ assays. Increasing EPSF6 concentrations from 100 to 1,500 μg/mL enhanced antioxidant activity in 60 min (Alharbi et al., 2023). Also, the *Achromobacter piechaudii* NRC2 EPS fraction exhibits strong anti-cyclooxygenase and antioxidant properties (Asker et al., 2015). Liu et al. (2019) identified two free radical-scavenging polysaccharides from *Floccularia luteovirens* fermentation fluid.

Environmental stresses can trigger microorganisms to form protective biofilms by adhering to surfaces. These biofilm-forming pathogenic bacteria are responsible for antibiotic resistance, chronic infections, shielding from the host’s immune defences, and recurrent infections due to their ability to reside on medical devices and surfaces (Shree et al., 2023). The resulting biofilms pose significant threats to food safety and public health as they persist on surfaces and resist conventional antibiotics (Abebe, 2020). Biofilm-mediated infections are challenging to treat due to the biofilm matrix’s impenetrability and embedded bacteria’s resistance mechanisms. Therefore, new approaches are needed to prevent biofilm formation or eradicate established biofilms in clinical and industrial settings (Sharma et al., 2023).

The microbial EPSs exhibited significant inhibitory effects on the adhesion, colonization, and growth of diverse Gram-positive microbes. Bacteria from the *Lactobacillus*, *Streptococcus*, *Bacillus*, *Weissella*, *Leuconostoc*, and *Limosilactobacillus* genera biosynthesize these bacteriostatic or bactericidal EPSs (Abdalla et al., 2021). While some microbial EPSs exhibited broad-spectrum responses, others only acted in one particular species (Angelin and Kavitha, 2020). For example, an EPS synthesized by *Bifidobacterium longum* demonstrated a unique mechanism of action against pathogens. Instead of conventionally inhibiting microbial growth, this EPS impedes the cell division processes in several bacterial pathogens by disrupting cell replication and proliferation rather than suppressing growth (Wu et al., 2010). An EPS-synthesized *Lactobacillus rhamnosus* showed potent *in vitro* antibacterial species effects against the pathogenic strains *Escherichia coli* and *Salmonella typhimurium* (Riaz Rajoka et al., 2018). Another EPS from *Lactobacillus* sp. Ca6 displayed inhibited *Micrococcus luteus* with an inhibition zone of 14 mm and *Salmonella enterica* with a 10 mm zone (Trabelsi et al., 2017).

The search for novel EPS-producing bacteria is crucial, given the limited number of bacterial strains documented as EPS producers. Moreover, it has been reported that G+ve *Bacillus* species are EPS-potent generators, and their metabolites are currently recognized as promising pharmaceutical natural substances (Angelin and Kavitha, 2020).

Based on the remarkable practicality of microbial EPSs and the ongoing endeavours to uncover and investigate new microbial bioactive exopolysaccharides. Therefore, the main goals of this work are to isolate novel EPS-producing marine bacteria from the Red Sea and fully characterize their extracted bioactive EPS compounds. Our study lies in identifying new marine bacterial strains as sources of antimicrobial and antioxidant EPS agents, widening the search for new therapeutic EPS compounds and aiding future drug discovery efforts. Subsequently, the EPS extraction, purification, and chemical characterization from selected isolates will be performed using precipitation, UV–VIS, and FT-IR HPLC techniques. Furthermore, the therapeutic potential of purified EPS will be evaluated by investigating their antioxidant using assays like DPPH and ABTS^+^, TAC, FRAP, antimicrobial screening by agar diffusion, MIC/MBC determination, and microtiter plate biofilm quantification at different sub-MBCs. The antifungal effects against yeasts and filamentous fungi will be assessed by measuring parameters such as inhibition zone, MIC, and MFC.

## 2 Materials and methods

### 2.1 Selective isolation, genetic classification, and phylogenetic evaluation of the bacterial strains

A seawater specimen of 500 mL was obtained from the western shores of the Red Sea in Saudi Arabia in November 2023. The sample was taken from the sea’s surface utilizing a sterile flask and transported to the laboratory in a chilled insulated casing at 4°C. In the laboratory, serial dilution and marine media were prepared by introducing the subsequent components while maintaining their volumes and concentrations constant: glucose (20 g), CaCO_3_ (1.0 g), NH_4_NO_3_ (0.8 g), KH_2_PO_4_ (0.05 g), K_2_HPO_4_ (0.6 g), MgSO_4_.7H_2_O (0.05 g), MnSO_4_.4H_2_O (0.1 g), and yeast extract (0.1 g) were added to 750 mL of seawater to make 1 L. The serial dilution approach was used to collect and isolate bacterial specimens from the seawater samples (Hayakawa and Nonomura, 1987). The bacterial strains were selectively chosen based on their maximal EPS generation rate and culture growth parameters. For bacterial genetic classification, the following primers were used: The forward primer 5′-TCCGTAGGTGAACTTTGCGG 3′, and the reverse primer was 5′-TCCTCCGCTTATTGATATGC-3′, following the 16S rRNA sequence was performed for further phylogenetic analysis (Tamura et al., 2011). The obtained DNA sequence was compared to the NCBI’s GenBank database using the BLAST tool. The degree of sequence similarity between the isolate’s sequence and those in the database was then evaluated by a sequence alignment.

### 2.2 Production, characterization, and structural components analysis of the EPS

The strain R2 was chosen as the probable candidate for EPS production. The last stage was incorporating the fermentation medium broth, as outlined in the study by Liu et al. (2010).

The bacterial isolate R2 was grown in a medium containing yeast extract (2 g/L), sucrose (20 g/L), and peptone (4 g/L). These components were added to 750 mL of seawater and then brought up to 1 L. After cultivation, the bacteria were separated by centrifugation at 4000 rpm at 4°C for 30 min. TCA (10%) was introduced to remove proteins, and the mixture was kept at 4°C overnight. The solution was centrifuged again for 20 min at 5000 rpm, retaining the supernatant liquid separately. The liquid supernatant pH was then adjusted to 7 using NaOH solution. Subsequently, the supernatant was precipitated with cold C_2_H_5_OH and centrifuged. The residue was redissolved, dialyzed for 72 h, and fractionally precipitated with 4 successive increasing volumes of C_2_H_5_OH. An analysis of the UV absorption spectra between 200 and 800 nm to identify the presence of nucleic acids and proteins (Rajivgandhi et al., 2021). The EPS FTIR spectra were analyzed to identify characteristic peaks corresponding to key functional groups present in the extracted polysaccharides. The EPS sample was prepared for FTIR analysis by grinding 2.0 mg of EPS with 200 mg of KBr to produce KBr pellets. The FTIR spectra were then examined using the Bruker Vector 22 FTIR spectrophotometer unit (Kadaikunnan et al., 2021). The colourimetric technique outlined by Filisetti-Cozzi and Carpita (1991) was used to identify uronic acid in the EPS. Sulfate concentrations were determined using Garrido’s technique (Garrido, 1964). The specimen’s monosaccharide content was measured using the Rajivgandhi et al. approach. To start, acid hydrolysis was performed by hydrolyzing a known quantity of EPS (15 mg) with HCOOH (88%) in a sealed vessel at 100°C for 5 h. Afterward, the hydrolysate was quantitatively transferred to a crucible, and HCOOH evaporated to dryness under a vacuum at 40°C. The hydrolysate was then washed with dH_2_O and concentrated under vacuum after repeatedly evaporating to eliminate the formic acid. The sample was frozen in a sealed vial for later analysis. Next, HPLC was used to separate and quantify the EPS hydrolysate by analyzing the mono sugars on an Agilent Pack series 1,200 instrument equipped with an Aminex carbohydrate HP-87C column (300 mm × 7.8 mm). The mobile phase was deionized H_2_O at a flow rate of 1 mL/min. Peaks were identified by comparing retention times to known reference standards. Concentrations of sugars were calculated from retention times and peak areas using Agilent Packard data analysis (Rajivgandhi et al., 2018).

### 2.3 Antioxidant evaluation of the isolated EPS

#### 2.3.1 DPPH screening

The assessment of the antioxidative capacity of the EPS was conducted at different concentrations (ranging from 1.95 to 1,000 μg/mL) utilizing the approach outlined by Brand-Williams et al. (1995). The spectrophotometer used in this test was the UV–VIS Milton Roy model. It was employed to quantify the absorbance at a specific wavelength of 517 nm. Ascorbic acid was applied as the reference standard during the experimental process, and the testing procedure was done in triplicate. The IC_50_ value of the EPS was obtained through the construction of a logarithmic dose-inhibition curve.


$$\mathrm{DPPH}\;\mathrm{inhibition}\;\left(\%\right)=[\left({\mathrm{Abs}}_{\mathrm{control}}-{\mathrm{Abs}}_{\mathrm{sample}}/{\mathrm{Abs}}_{\mathrm{control}}\right]\times 100$$

#### 2.3.2 Quantification of EPS total antioxidant capacity

The EPS was quantitatively examined using the phosphomolybdenum method and spectrophotometric analysis, following Prieto et al. (1999). Utilizing a microtiter plate reader (Biotek ELX800; Biotek, Winooski, VT, United States), the absorbance at 630 nm was quantified. The values were calculated using the ascorbic acid equivalent (AAE) unit, represented in μg/mg of the tested EPS, according to Lahmass et al. (2018).

#### 2.3.3 Determining EPS ferric reducing antioxidant power through potassium ferricyanide reduction

The potassium ferricyanide trichloroacetic acid method described by Benzie and Strain (1996) was used to examine the effect of solvent polarity on the total reducing capability of the EPS. The measurements were carried out with a microtiter plate reader (Biotek ELX800; Biotek, Winooski, VT, United States) at a wavelength of 630 nm. Ascorbic acid at a 1 mg/mL dosage was utilized as the positive control in the experiment, while DMSO acted as the negative control. The results were measured and reported as ascorbic acid equivalent (AAE) μg/mg of EPS.

#### 2.3.4 ABTS^+^ scavenging assessment of EPS

The ABTS^+^ assay was performed based on Re et al. with some modifications. ABTS was dissolved in water at 7 mM, reacted with 2.45 mM potassium persulfate, and kept at room temperature in the dark to generate the ABTS radical cation ABTS^+^. The ABTS^+^ cation solution was diluted with water to an absorbance of 0.70 at 734 nm. The reaction mixture consisted of 0.07 mL of EPSR2 and 3 mL of the ABTS radical solution; EPSR2 was then incubated with the diluted ABTS^+^ solution for 6 min before measuring absorbance at 734 nm (González-Palma et al., 2016).


$${\mathrm{ABTS}}^{+}\;\mathrm{inhibition}\;\left(\%\right)=[\left({\mathrm{Abs}}_{\mathrm{control}}-{\mathrm{Abs}}_{\mathrm{sample}}/{\mathrm{Abs}}_{\mathrm{control}}\right]\times 100$$

### 2.4 Antibacterial and antifungal screening of the EPS

Using the agar well diffusion technique, the antibacterial properties of the EPS were examined against a broad spectrum of bacterial species on Mueller-Hinton agar media and Sabouraud dextrose agar for fungi from the ATCC collection. The presented G+ve bacterial strains include *Bacillus Subtilis* (ATCC 6633), *Staphylococcus aureus* (ATCC 6538), and *Enterococcus faecalis* (ATCC 29212). The G−ve bacteria tested in this study were *Escherichia coli* (ATCC 8739), *K. pneumoniae* (ATCC13883), *Salmonella typhi* (ATCC 6539), and *Pseudomonas aeruginosa* (ATCC 90274). The tested fungi were; *Aspergillus niger* (AUMC 14260), *Mucor circinelloid* (AUMMC 11656), *Trichoderma harzianum* (AUMC 5408) *Penicillium glabrum* (OP694171) (AUMC15597) and *Candida albicans* (ATCC 10221).

After the agar had been dried for 15 min, the Microbial suspension was spread in three directions, and a sterile cork was used to make a 6 mm hole in the plate. EPS and Gentamicin were dissolved in MDSO at a 10 mg/mL dosage. Gentamicin was employed as the control medication for bacterial inhibition screening. In comparison, Fluconazole served as the antifungal control in this test; 10 mg/mL units of EPS were put into the well. After disposal, the plates were incubated for two days for bacteria (Magaldi et al., 2004) and 16 to 24 h for (*Mucor circinelloid*es), 24 h for (*A. niger*), 48 h for (*C. albicans, T. harzianum and P. glabrum*) (Espinel-Ingroff et al., 2007). When a discernible decline in growth occurred, the diameters of the inhibition zones surrounding the wells were measured to the closest full mm (Espinel-Ingroff et al., 2011). Subsequently, the minimum bactericidal concentrations (MBCs), minimum inhibitory concentrations (MICs), and minimum fungicidal concentrations (MFC) were investigated following the recommendations established by the Clinical and Laboratory Standards Institute (CLSI) (Brown, 1988).

### 2.5 Antibiofilm evaluation of the EPS

EPS’s influence on biofilm formation was measured in 96-well polystyrene flat-bottom plates. In summary, trypticase soy yeast broth (TSY) in a volume of 300 𝜇L was cultivated with 75, 50, and 25% of MBC of the previously examined organisms up to a final concentration of 10^6^ (CFU/mL). Following two days of incubation at 37°C, the biofilm on the plates was stained for fifteen minutes using an aqueous solution containing 0.1% crystal violet. Any remaining stain on the plate was removed using sterile dH_2_O following the incubation time. 250 𝜇L of 95% C_2_H_5_OH was added to each well to dissolve the dye attached to the cells. After 15 min and using a microplate reader, absorbance was measured at 570 nm after 15 min (Antunes et al., 2010).

### 2.6 Statistical analysis

Triplicates were used for all tests. The results are shown as mean ± SD; data were evaluated using one-way ANOVA and Tukey *post hoc* test. The SPSS program (V27) applied the *T*-test for comparisons, *n* = 3, *p* ≤ 0.05.

## 3 Results

### 3.1 Characterization and identification of EPS marine bacterial strain R2 as *Bacillus licheniformis* LHG166 using phenotypic and genotypic methods

A thorough collection of 10 bacterial isolates originated from marine sand samples acquired from the Red Sea and afterwards submitted to a screening process to test their ability to produce EPS. The screening process involved the evaluation of cultural growth characteristics, morphological aspects, and EPS production yield quantification. The marine bacteria strain R2 demonstrated the highest production of EPS (7.22 g/L). The production of EPS primarily consisted of a substantial fraction, accounting for 91.81% of 3-volume ethanol. Classical microbiology examination indicated G+ve, short rod-shaped bacteria. Colony characteristics included a smooth surface, rough texture, and pale-yellow colour. The elevation was flat, and the edges were entire. The whole colony appeared irregular, with no pigmentation on large colonies. The opacity was opaque and grew under anaerobic conditions (Supplementary Table S1). Additional biochemical tests revealed positive results for starch hydrolysis, catalase production, urease activity, Voges-Proskauer, and Simmons citrate tests. Nitrate reduction was also positive. Carbohydrate fermentation was positive for glucose, maltose, sucrose, lactose, arabinose, and mannitol (Supplementary Table S2).

**Figure 1:**
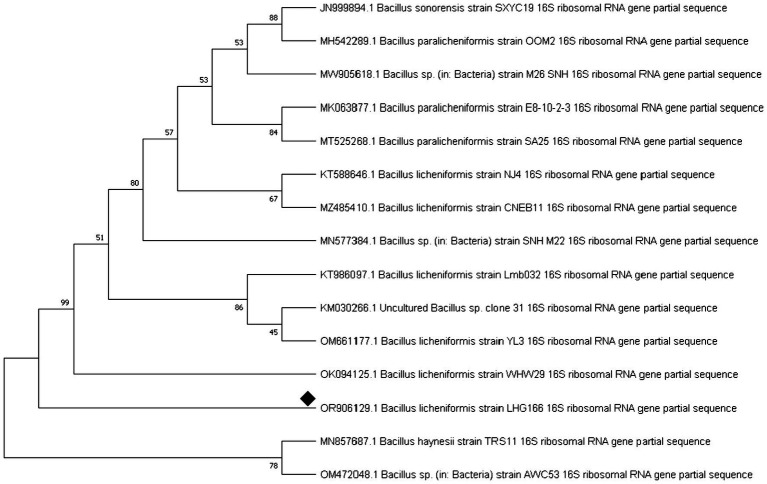
Neighbour phylogenetic tree analysis of *Bacillus licheniformis* LHG166 16srRNA.

Next, the bacterial 16S rRNA gene was sequenced, and a phylogenetic tree was constructed based on comparing sequences highly similar to the rRNA genes of the bacteria. Successful tree assembly was demonstrated by finding an alignment between the obtained rRNA gene sequences and those of *Bacillus licheniformis* (Figure 1). The identification of *Bacillus licheniformis* LHG166 was verified by the accession number (OR906129.1). After BLAST analysis, the DNA sequence was submitted to NCBI GenBank.

### 3.2 Structural elucidation of EPSR2 from *Bacillus licheniformis* LHG166 through spectroscopic and chromatographic techniques

**Figure 2:**
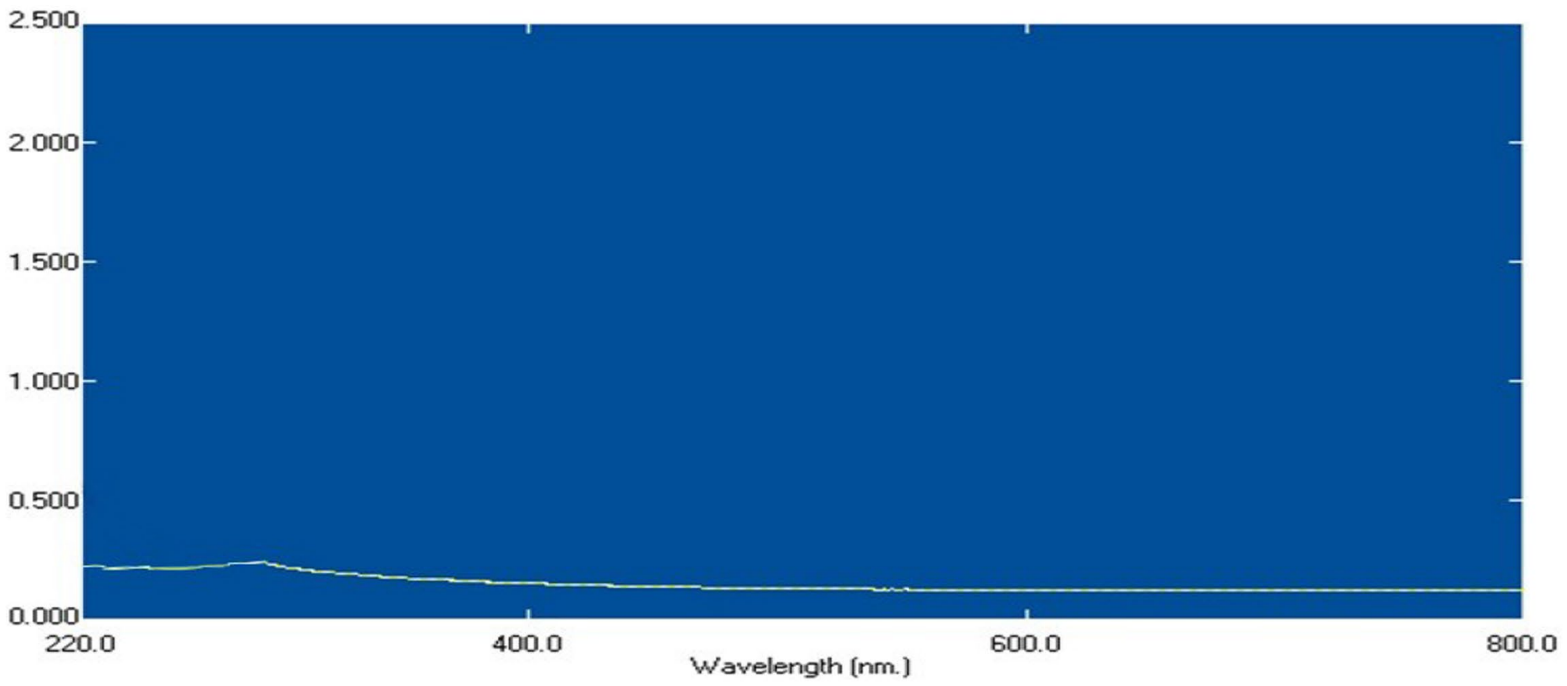
U.V spectrum of EPSR2 isolated from *Bacillus licheniformis* LHG166.

*Bacillus licheniformis* LHG166 was chosen as the best candidate for exopolysaccharide (EPSR2) synthesis because it generated a yield of 7.22 g/L. Next, a fractionation and precipitation purification procedure was applied to the unrefined residue. Following a three-day treatment with deionized H_2_O, the EPSR2 sample was filtered through a membrane with a pore size of 100 microns. A gradual treatment with cold C_2_H_5_OH was administered to the dialysis-affected EPSR2, leading to fractional precipitation. Three different ethanol precipitation methods were employed to obtain the EPSR2 core fraction, which accounted for 91.81% of the total fraction from the initial crude EPS sample. The resultant fraction was subjected to 200–800 nm UV absorption spectra (Figure 2). The composition of EPSR2 was found to consist of uronic acid (48.11%), sulfate (17.40%), and N-acetyl glucose amine (6.09%).

**Figure 3:**
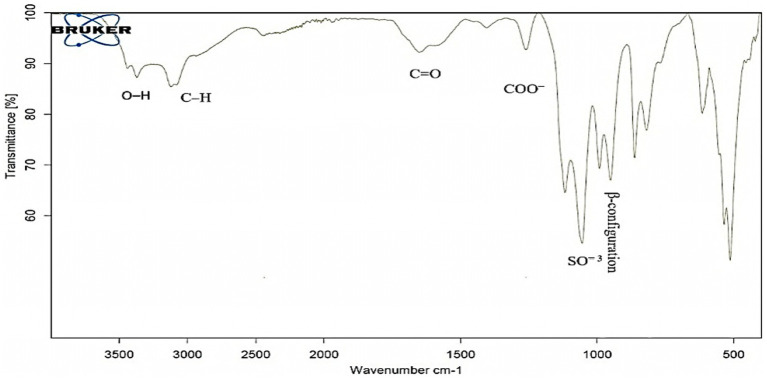
FT-IR spectrum of EPSR2 showing main functional groups.

As evidenced by the FT-IR, the broad characteristic peak at 3372.05 cm^−1^ was assigned to OH^−1^ stretching vibration. The band at 3123.80 cm^−1^ correlated with the C-H stretching vibration in the sugar ring. Also, the presence of absorption at 1652.16 cm^−1^ is referred to as C=O. While COO^−^ vibration at 1260.61 cm^−1^. The band at 1054.91 cm^−1^ indicated the SO^=3^, and the present was characteristic absorption at 862.88 cm^−1^ arising from β-configuration of the sugar units (Figure 3).

**Figure 4:**
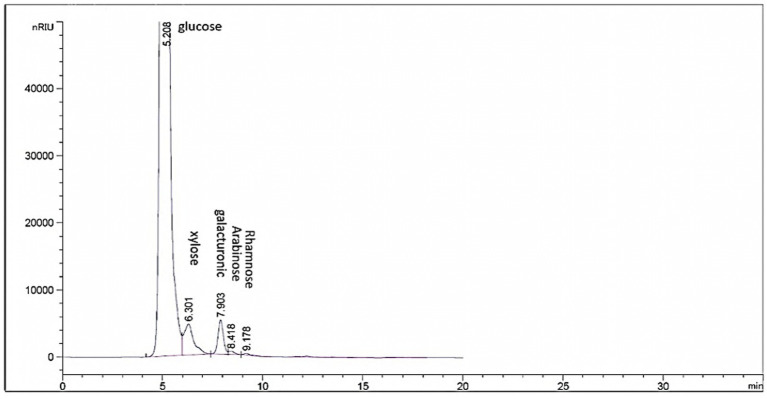
HPLC chromatogram of EPSR2 showing the monosaccharide molar ratios.

HPLC chromatogram of EPSR2 revealed the monosaccharides fractions (Glucose: xylose: galacturonic acid: arabinose: Rhamnose) with molar ratio: 1: 0.5: 2: 0.5: 0.5, respectively (Figure 4).

### 3.3 Antioxidant evaluation of EPSR2 by DPPH, ABTS^+^, TAC, and FRAP

**Figure 5:**
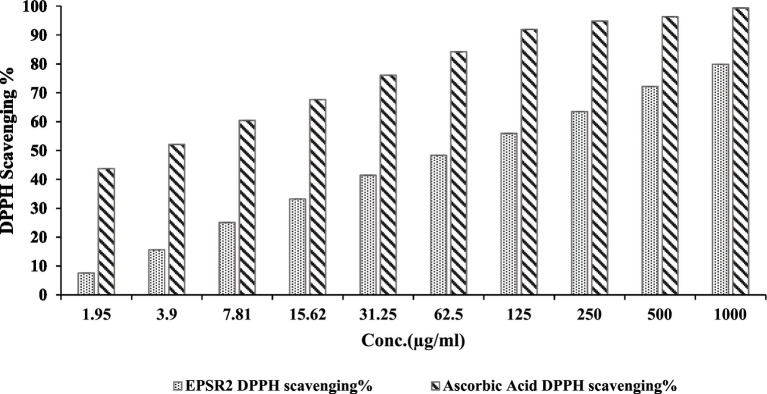
Dose–dependent concentrations of EPSR2 (1.95 to 1,000 μg/mL) DPPH radical scavenging % vs. ascorbic acid. Results represented as mean ± SD. One-way ANOVA (*n* = 3, *p* ≤ 0.05).

In the DPPH radical scavenging experiment, EPSR2 demonstrated concentration-dependent antioxidant capacity. EPSR2 inhibited DPPH radicals by 79.9% at the maximum measured dose of 1,000 g/mL (meanOD = 0.310). The One-way ANOVA results demonstrated that EPSR2’s DPPH scavenging was statistically significant (*p* < 0.05) across all concentration levels. Through extrapolation of the dose–response curve, the IC_50_ for EPSR2 was estimated to be 72.89 μg/mL (Figure 5). The conventional antioxidant ascorbic acid had an IC_50_ of 2.52 μg/mL. The t-test revealed that ascorbic acid had considerably higher radical scavenging ability than EPSR2 at similar doses (*p* < 0.05). EPSR2 had concentration-dependent antioxidant activity, although IC_50_ values and t-test statistics showed that it was lower than ascorbic acid at equal doses.

**Figure 6:**
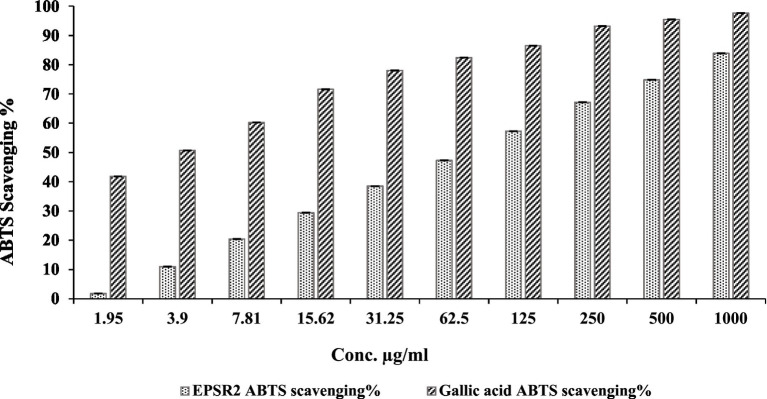
Concentration-dependent ABTS^+^ scavenging % by EPSR2 (1.95 to 1,000 μg/mL) vs. the standard Gallic acid (*n* = 3, *p* < 0.05, mean ± SD, one-way ANOVA).

The ABTS^+^ radical scavenging assay demonstrated that EPSR2 has dose-dependent antioxidant activity, though moderate in potency. Specifically, EPSR2 exhibited increasing scavenging percentages from 0 to 83.9% at concentrations ranging from 0 to 1,000 μg/mL, with an IC_50_ of 74.52 μg/mL compared to 2.54 μg/mL for Gallic Acid. At low concentrations of 1.95 and 3.9 μg/mL, EPSR2 showed minimal effects, with only 1.8 and 11.0% scavenging, respectively. However, the scavenging steadily augmented in a dose-responsive manner to 20.4% at 7.81 μg/mL and further to 74.8% at 500 μg/mL. The highest tested concentration of 1,000 μg/mL resulted in 83.9% scavenging compared to 97.6% for Gallic acid, which served as the control at the same tested concentration (Figure 6).

The TAC assay was then performed in triplicates, and EPSR2 showed a mean TAC of 106.07 μg/mg (AAE) (Table 1), demonstrating that EPS possesses appreciable *in vitro* antioxidant capacity comparable to Ascorbic acid control. The FRAP method evaluated the reducing antioxidant ability of ESP to reduce ferric (Fe^3+^) to ferrous (Fe^2+^) ions (Supplementary Tables S3, S4). The assay was performed in triplicates, and the mean FRAP value obtained for ESPR2 was 60.1 μg AAE/mg (Table 1).

**Table 1:** TAC and FRAP values of EPSR2.

EPSR2 (AAE) μg/mg	TAC (equivalent (AAE) μg/mg) mean ± SD	FRAP (equivalent (AAE) μg/mg) mean ± SD
	106.07 ± 0.221	60.1 ± 0.8

Values expressed as mean ± SD μg/mg (AAE).

### 3.4 Evaluating the antimycotic effects of EPSR2 against filamentous fungi and yeasts

Of all the fungi tested, the yeast *Candida albicans* displayed the greatest susceptibility to EPSR2. *C. albicans* showed an inhibition zone of 28 mm, MIC of 7.8 μg/mL, and MFC of 15.62 μg/mL, indicating potent antifungal activity. In contrast, the fungi *Mucor circinelloides* and *Trichoderma harzianum* were highly resistant to EPSR2. No inhibitory activity was detected against these fungi, even at the highest concentrations tested (Supplementary Figure S1).

At the highest concentration of 75% of MBC, EPSR2 showed 96.03% inhibition of biofilm formation compared to the untreated control. Even at the lowest dose of 25% of MBC, EPSR2 still reduced *C. albicans* biofilm mass by 86.20%. As the concentration of EPSR2 decreased from 75 to 50 to 25% of MBC, its anti-biofilm potency also declined dose-dependently. At 50% of MBC, EPSR2 inhibited 92.16% of biofilm, while at 25% of MBC, the inhibition dropped to 86.20% (Table 2).

**Table 2:** EPSR2 antibiofilm activity against *Candida albicans* at 25, 50, and 75% MBC.

EPSR2 – MBC% of *C. albicans*	EPSR2 – anti-biofilm activity %
Blank (media only)	–
Media organism (cont.)	–
25% of MBC	86.20
50% of MBC	92.16
75% of MBC	96.03

The fungi *Aspergillus niger* and *Penicillium glabrum* demonstrated moderate susceptibility to EPSR2. *A. niger* had an 18 mm zone of inhibition, 250 μg/mL MIC, and 1,000 μg/mL MFC. *P. glabrum* showed slightly better activity, with a 21 mm zone, 125 μg/mL MIC, and 1,000 μg/mL MFC. However, their susceptibility was much lower than that of the yeast *C. albicans* (Table 3).

**Table 3:** Comparative susceptibility testing of EPSR2 against different filamentous fungi and yeasts.

Tested filamentous fungi/yeasts	EPSR2 inhibition zone (mm)	Fluconazole control (mm)	MIC (μg/ml)	MFC (μg/ml)
*Aspergillus niger* (AUMC 14260)	18 ± 0.1	36 ± 0.1	250	1,000
*Mucor circinelloides* (AUMMC 11656)	0.9 ± 0.1	23 ± 0.2		–
*Trichoderma harzianum* (AUMC 5408)	–	32 ± 0.1	–	–
*Penicillium glabrum* (OP694171) (AUMC15597)	21 ± 0.2	38 ± 0.1	125	1,000
*Candida albicans* (ATCC 10221)	28 ± 0.1	26 ± 0.3	7.8	15.62

### 3.5 Profiling the antimicrobial effects of *Bacillus licheniformis* LHG166 EPSR2 against G+ve and G−ve bacteria

**Figure 7:**
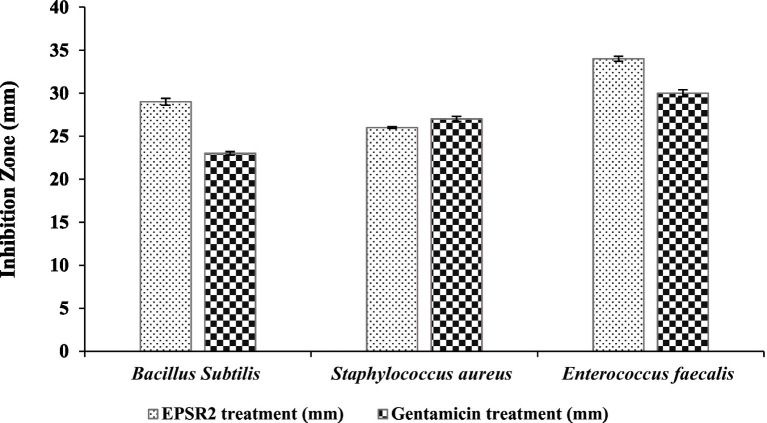
The antibacterial effect of EPSR2 against G+ve pathogenic bacteria represented as inhibition zone (mm).

EPSR2 exhibited broad-spectrum antibacterial activity against G+ve and G−ve bacteria in the agar well diffusion assay. Among the Gram positives, EPS showed the largest inhibition zone of 34 ± 0.3 mm against *Enterococcus faecalis* compared to 30 ± 0.4 of Gentamicin. In contrast, *Staphylococcus aureus* had a minor inhibition zone of 26 ± 0.1 mm compared to 27 ± 0.3 of Gentamicin (Figure 7; Supplementary Figure S2).

For MICs, the values ranged from 7.8 to 31.25 μg/mL. *Bacillus subtilis* had the highest MIC value at 31.25 μg/mL, while *Enterococcus faecalis* had the lowest MIC of 7.8 μg/mL. Regarding MBCs, values ranged from 15.62 to 62.5 μg/mL. The highest MBC value was 62.5 μg/mL observed against *Bacillus subtilis*, while the lowest MBC was 15.62 μg/mL with *Staphylococcus aureus* and *Enterococcus faecalis* (Table 4). The MBC/MIC ratio was 2 for both *B. subtilis* and *Enterococcus faecalis. For Staphylococcus aureus,* the ratio was 1, indicating the bactericidal effect of EPSR2 against all tested G+ve bacteria.

**Table 4:** The antibacterial potential of EPSR2 is represented as inhibition zones (mm), MIC, and MBC against G+ve bacteria.

Pathogenic G+ve bacteria	EPSR2 (mm)	Gentamicin (control)	MIC (μg/ml)	MBC (μg/ml)	MBC/MIC ratio
*Bacillus subtilis* (ATCC 6633)	29 ± 0.4	23 ± 0.2	31.25	62.5	2
*Staphylococcus aureus* (ATCC 6538)	26 ± 0.1	27 ± 0.3	15.62	15.62	1
*Enterococcus faecalis* (ATCC 29212)	34 ± 0.3	30 ± 0.4	7.8	15.62	2

**Figure 8:**
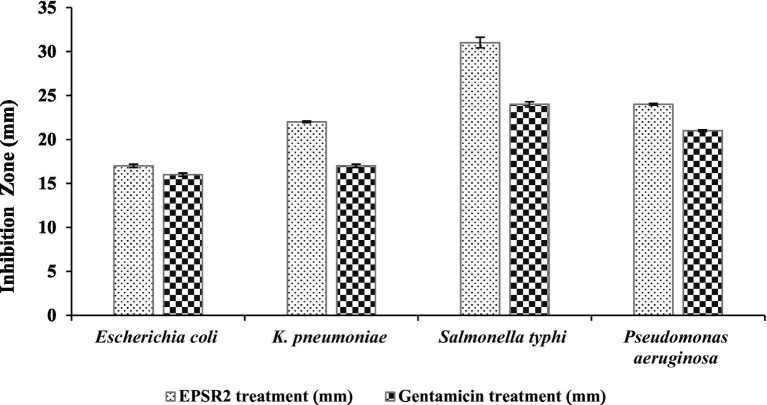
The antibacterial effect of EPSR2 against G−ve pathogenic bacteria represented as inhibition zone (mm).

Based on the measured inhibition zone diameters, EPSR2 displayed inhibitory effects against all four G−ve bacterial pathogens. The highest inhibition zone of 31 ± 0.6 mm was seen against *Salmonella typhi* compared to 24 ± 0.3 mm for Gentamicin. The lowest inhibition was observed against *Escherichia coli*, with EPSR2 showing an inhibition zone of 17 ± 0.2 mm, similar to the 16 ± 0.2 mm zone of Gentamicin. EPSR2 demonstrated comparable or larger inhibition zones than the antibiotic gentamicin against all the tested strains. Specifically, EPSR2 had a moderately higher inhibition zone of 22 ± 0.1 mm versus 17 ± 0.2 mm for Gentamicin against *Klebsiella pneumoniae*. Additionally, EPSR2 exhibited slightly greater inhibition of *Pseudomonas aeruginosa* growth with a 24 ± 0.1 mm zone versus 21 ± 0.1 mm for Gentamicin (Figure 8; Supplementary Figure S3).

The lowest MIC of 15.62 μg/mL was seen against *Salmonella typhi*, most susceptible to growth inhibition by EPSR2. Its MBC was 31.25 μg/mL, and the MBC/MIC ratio was 2, suggesting EPSR2 has bactericidal effects against *S. typhi*. In contrast, EPSR2 displayed the highest MIC and MBC values of 250 μg/mL against *Escherichia coli*, meaning this strain was the least sensitive to EPSR2’s antimicrobial effects. However, the MBC/MIC ratio was 1, indicating bactericidal activity against *E. coli*. *Klebsiella pneumoniae* showed an intermediate level of susceptibility, with an EPSR2 MIC of 125 μg/mL, MBC of 125 μg/mL, and MBC/MIC ratio of 1, consistent with bactericidal action. *Pseudomonas aeruginosa* was also moderately susceptible based on a MIC of 31.25 μg/mL, a high MBC of 125 μg/mL, and an MBC/MIC ratio of 4, which indicates EPSR2 bactericidal impact (Table 5).

**Table 5:** The antibacterial potential of EPSR2 is represented as inhibition zones (mm), MIC, and MBC against G−ve ATCC bacterial pathogens.

Pathogenic G−ve bacteria	EPSR2 (mm)	Gentamicin (control)	MIC (μg/ml)	MBC (μg/ml)	MBC/MIC ratio
*Escherichia coli* (ATCC 8739)	17 ± 0.2	16 ± 0.2	250	250	1
*K. pneumoniae* (ATCC13883)	22 ± 0.1	17 ± 0.2	125	125	1
*Salmonella typhi* (ATCC 6539)	0.6 ± 31	24 ± 0.3	15.62	31.25	2
*Pseudomonas aeruginosa* (ATCC90274)	24 ± 0.1	21 ± 0.1	31.25	125	4

The comparable or superior inhibition zones of EPSR2 relative to the standard Gentamicin highlight its potential as a novel antibacterial agent against Gram-positive and Gram-negative bacterial pathogens.

### 3.6 Profiling the anti-biofilm activity of EPSR2 at sub-MBCs against G+ve and G−ve ATCC pathogenic bacterial strains

**Figure 9:**
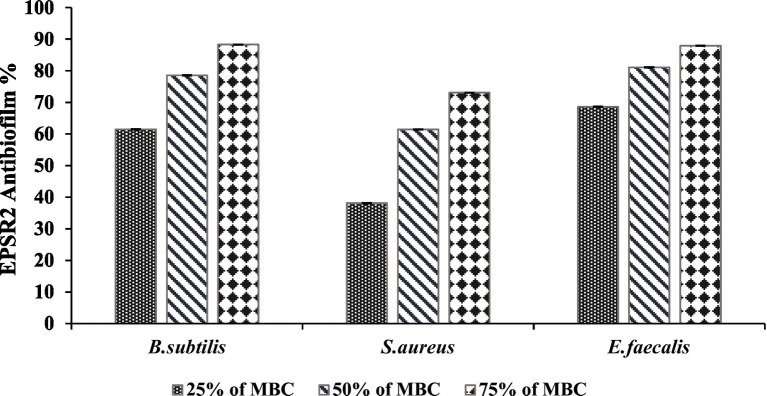
Antibiofilm activity of EPSR2 against different G+ve ATCC bacteria at different %MBC.

EPSR2 exhibited concentration-dependent anti-biofilm effects against the tested G+ve bacteria *B. subtilis*, *S. aureus*, and *E. faecalis*. At 75% of MBC, EPSR2 showed the highest anti-biofilm activity against *B. subtilis*, inhibiting 88.30% of biofilm formation (Table 6). In contrast, the lowest biofilm inhibition of 73.06% at 75% of MBC was observed with *S. aureus*. As the concentration of EPSR2 decreased, so did its anti-biofilm potency. At 25% of MBC, the biofilm inhibition dropped to 68.61% for *E. faecalis* and 61.46% for *B. subtilis*. At the same time, *S. aureus* displayed an intermediate susceptibility profile to the anti-biofilm effects of EPSR2. At 50% MBC, its antibiofilm inhibition was 61.42%, whereas at 25%, its antibiofilm activity was 38.09% (Figure 9; Supplementary Table S5).

**Table 6:** EPSR2 antibiofilm activity against *Bacillus subtilis* at 25, 50, and 75% MBC.

EPSR2 – MBC% of *B. subtilis*	EPSR2 – anti-biofilm activity %
Blank (media only)	–
Media organism (cont.)	–
25% of MBC	61.46
50% of MBC	78.54
75% of MBC	88.30

In summary, EPSR2 demonstrated the greatest anti-biofilm potential against *B. subtilis*, with 88.3% inhibition at 75% of MBC, compared to the lowest inhibition of 73.06% seen with *S. aureus* at the same concentration. The declining anti-biofilm activity at sub-inhibitory EPSR2 concentrations highlights the concentration-dependent nature of its effects against G+ve bacteria.

**Figure 10:**
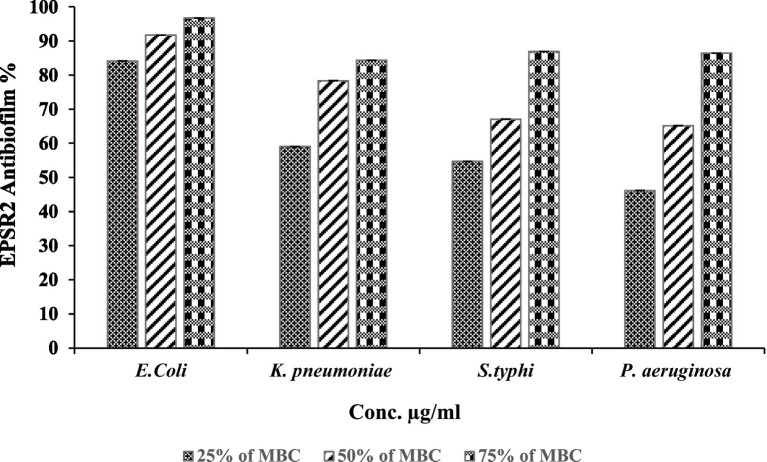
Antibiofilm activity of EPSR2 against different G−ve ATCC bacteria at different %MBC.

Concerning G−ve biofilm inhibition, EPSR2 exhibited potent, dose-dependent anti-biofilm formation against all pathogenic Gram-negatives; at 75% of MBC, EPSR2 displayed the highest anti-biofilm activity of 96.63% against *E. coli* (Table 7), followed by 86.91% against *S. typhi*, 86.45% against *P. aeruginosa*, and 84.36% against *K. pneumonia*. Even at the lowest dose of 25% MBC, EPSR2 reduced biofilm formation by 84.13% in *E. coli*, 58.99% in *K. pneumoniae*, 54.67% in *S. typhi*, and 46.17% in *P. aeruginosa*. *E. coli* was the most susceptible to the anti-biofilm effects of EPSR2 across the tested concentrations. In contrast, *P. aeruginosa* was relatively the most resistant, requiring higher EPSR2 doses to achieve biofilm reduction comparable to the other bacteria (Figure 10; Supplementary Table S6).

**Table 7:** EPSR2 antibiofilm activity against *Escherichia coli* at 25, 50, and 75% MBC.

EPSR2 – MBC% of *E. coli*	EPSR2 – anti-biofilm activity %
Blank (media only)	–
Media organism (cont.)	–
25% of MBC	84.13
50% of MBC	91.7
75% of MBC	96.63

## 4 Discussion

Microbes are more potent and cheaper sources of EPSs compared to plants. The high growth rate and easy manipulation of microbes enable efficient and scalable production of EPSs. Additionally, growing microbes in inexpensive media reduces overall production costs. Their lower space requirements also make large-scale fermentation more feasible. Therefore, microbial EPSs are promising alternative sources that can compete with conventional plant-derived EPSs in terms of cost and volume (Yildiz and Karatas, 2018; Osemwegie et al., 2020; Ibrahim et al., 2022). This work focuses on tapping into the pharmacologic potential of marine bacteria as a source of antimicrobial and antioxidant EPS metabolites that could substitute traditional antibiotics and provide effective natural product-based therapeutics.

Out of 10 bacterial isolates screened, a marine bacterial isolate, R2, was isolated from the Red Sea with an output of 7.22 g/L of EPSR2. The R2 isolate was then identified as a rod-shaped, gram-positive bacteria with pale yellow colonies (Supplementary Tables S1, S2). Its 16S rRNA gene sequence closely matched that of *Bacillus licheniformis* LHG166 regarding phylogeny (Figure 1). The EPSR2 main functional groups, as evidenced by FT-IR, were uronic acid (48.11%), sulfate (17.40%), and N-acetyl glucose amine (6.09%) (Figure 3). The HPLC chromatogram of EPSR2 displayed the fractions of monosaccharides (Glucose: xylose: galacturonic acid: arabinose: Rhamnose) with a molar ratio of 1: 0.5: 2: 0.5: 0.5 (Figure 4).

Several variables influence the quantity of EPS generated by microorganisms, such as the age and size of the inoculum, the medium’s composition, and the physical characteristics of the growing conditions. The extracellular synthesis and production of several metabolites, particularly polysaccharides, are significantly influenced by the pH level of the medium, temperature, aeration, and agitation rate (Kaur and Dey, 2023). Polysaccharides derived from various sources have distinct biological activity across several levels (Prasad and Purohit, 2023). Their responses rely upon the molecular weight (MW), monosaccharide content, and structural conformation (linkage and branching degree). For example, EPS from wild-type and mutant strains of *W. confusa* displayed distinct functional groups at five positions, each showing varying degrees of antioxidant capacity and different amounts of antibodies generated in mice treated by EPS (Adebayo-Tayo et al., 2018).

Furthermore, their functional groups have impacted microbial polysaccharides’ bioactivities. The position and amount of sulfate groups primarily influence the bioactivities of sulfated EPS. For example, compared to a native EPS, a sulfated EPS produced via sulfonation from *Lactobacillus plantarum* ZDY2013 exhibited increased antioxidant activity (Zhang et al., 2016). Also, the molecular weight and the monosaccharides in the EPS’s structure impacted the bioactivities of these EPSs. The low-weight EPS (70 × 10^3^ Da) extracted from *Weissella confusa* stimulated RAW264.7 cells and induced NO and cytokines production, while native EPS did not. Size or MW may affect molecule binding and penetration (Li et al., 2016). Therefore, EPSs with a lower weight MW may have stronger cell receptor binding and could easily penetrate a cell with superior bioactivities than the larger MW EPSs. The monosaccharide structure and molar ratio have also contributed to the bioactivities of such EPSs. For example, The *Lactobacillus reuteri* Mh-001 EPS fractions exhibited immunomodulatory properties. These EPS’s monosaccharide content ratios affected their anti-inflammatory qualities. The EPS fraction with the highest galactose content for macrophages showed the most potent anti-inflammatory efficacy (Chen et al., 2019). Concluding the compositions of monosaccharides may be linked to the immune cell surface receptors’ detection (Ren et al., 2017). The current explored EPSR2 isolated from *Bacillus licheniformis* LHG166 comprised of glucose: xylose: galacturonic acid: arabinose: Rhamnose and with sulfate (17.40%), uronic acid (48.11%), and N-acetylglucosamine (6.09%) residues.

EPSR2 was then tested as a natural microbial antioxidant by DPPH, FRAP, TAC, and ABTS^+^ assays. The saccharide demonstrated concentration-dependent antioxidant activity in DPPH radical scavenging, inhibiting 79.9% at 1000 μg/mL and IC_50_ at 72.89 μg/mL (Figure 5). A typical antioxidant, ascorbic acid, exhibited a decreased IC_50_ of 2.52 μg/mL and inhibited DPPH by 99.3% at 1000 μg/mL instead of 79.9% by the examined EPSR2. Both TAC and FRAP assays performed in triplicates confirmed the *in vitro* antioxidant potential of the EPS with mean values (106.07 ± 0.221 and 60.1 ± 0.8 μgAAE/mg), respectively (Table 1). In ABTS assay, EPSR2 has moderate, dose-dependent ABTS activity with an IC_50_ of 74.52 μg/mL. Minimal scavenging was observed at low concentrations of 1.95–3.9 μg/mL (1.8–11%) but steadily increased to 83.9% at the highest 1,000 μg/mL tested concentration (Figure 6).

Following our findings, EPSR5, an acidic microbial exopolysaccharide extracted from marine *Kocuria* sp., its highest DPPH radical scavenging of 98% was recorded after 120 min at a concentration of 2000 μg/mL (Alshawwa et al., 2022). Also, Another EPS isolated from *B. subtilis* AG4 exhibited 97.6% DPPH scavenging activity at 1500 μg/mL, IC_50_ = 300 μg/mL, and 64.8% H_2_O_2_ scavenging, IC_50_ = 1,500 μg/mL at the same tested concentration (Abdel-Wahab et al., 2022)—another EPS from *Bacillus* sp. LBP32 can reduce LPS-induced inflammation by inhibiting oxidative stress (Diao et al., 2014). Also, another polysaccharide, EPSR3, extracted from f *B. cereus*, had IC_50_ = 500 μg/mL after 60 min for DPPH, while H_2_O_2_ was 1,500 μg/mL after 15 min (Selim et al., 2022). A Streptomyces polysaccharide composed of mannose and glucose showed effective hydroxyl and DPPH radical scavenging and high Fe^2+^ chelation ability (Elnahas et al., 2017). Prior research found that added sulfate, acetyl, and phosphate groups can enhance polysaccharides’ *in vitro* antioxidant capacity. As in the case of HePS synthesized by *L. plantarum* (Zhang et al., 2016) and *L. lactis* subsp. lactis (Guo et al., 2013) improved the antioxidant potential of their EPS. In light of the abovementioned, the antioxidant of the explored negatively charged EPSR2 synthesized by *Bacillus licheniformis* LHG166 and the current studies demonstrate that bacterial EPSs can be a good source of natural antioxidants.

Monosaccharide composition, molecular weight, and functional groups could influence the EPS antioxidant activity. In addition, the extraction and purification methods used could play a role. Among the EPS with low molecular mass, the acidic polymers often showed stronger antioxidant activities than the neutral ones (Fernandes and Coimbra, 2023). Also, the antioxidant activity of EPS is related to the presence of hydroxyl groups and other functional groups that can stabilize free radicals. Negatively charged groups can generate an acidic environment that facilitates EPS hydrolysis, exposing more hydroxyl groups with strong antioxidant activity (Bai et al., 2022).

Next, EPSR2 was tested as an antifungal compound against filamentous moulds and yeasts. The key finding was that the yeast *Candida albicans* displayed the greatest susceptibility to the exopolysaccharide EPSR2, with high antifungal activity evidenced by a 28 mm inhibition zone (Supplementary Figure S1), MIC of 7.8 μg/mL, and MFC of 15.62 μg/mL. In contrast, the filamentous fungi *Mucor circinelloides* and *Trichoderma harzianum* were resistant to EPSR2 even at the highest tested concentrations. The fungi *Aspergillus niger* and *Penicillium glabrum* showed moderate EPSR2 susceptibility, with inhibition zones of 18–21 mm, MICs of 125–250 μg/mL, and MFCs of 1,000 μg/mL (Table 3). However, their antifungal sensitivity was far lower than the potent effects observed against *C. albicans*. The varied antifungal activity highlights the species-specific effects of EPSR2. Further evaluation of EPSR2 as an antifungal agent should focus on treating candidiasis and related yeast infections based on its high effectiveness against *C. albicans* (Table 2). Expanding testing against other pathogenic yeasts could reveal additional clinical applications.

Certain microbial EPSs demonstrate antifungal properties, particularly those with an overall negative charge. The negative charge enables enhanced electrostatic interactions with fungal cells. One example is the EPS produced by *L. rhamnosus* GG, which was shown to inhibit the hyphal formation of Candida species *in vitro* cell culture experiments. Additionally, the same EPS has been demonstrated to decrease the hyphal elongation of *C. albicans* in an *in vitro* gut model. The dextran produced by *Weissella confusa* significantly inhibits biofilm formation by *C. albicans* strain SC5314 (Abdalla et al., 2021). EPSs from other *Lactobacillus* species also exhibit antifungal effects against *Candida* species (Vazquez-Munoz and Dongari-Bagtzoglou, 2021). Another EPS extracted from *Bacillus licheniformis* strain Dahb1 demonstrated anti-biofilm activity against *C. albicans* (Abinaya et al., 2018). In another study, an EPS produced by *Bacillus cereus*, a plant growth-promoting rhizobacterium, inhibited mycelial growth of the fungal pathogen *Alternaria alternata* (Parvin et al., 2023).

The bacterial EPSR2 exhibited broad-spectrum antibacterial activity against both G+ve and G−ve pathogens in the study. The highest susceptibility was seen against the G+ve *Enterococcus faecalis* (Figure 7) and the G−ve *Salmonella typhi* (Figure 8). Among the Gram-positives, EPSR2 showed the greatest potency against *Enterococcus faecalis*, with the largest inhibition zone of 34 ± 0.3 mm and the lowest MIC of 7.8 μg/mL. *Staphylococcus aureus* was the least susceptible strain to EPSR2, with the smallest inhibition zone of 26 ± 0.1 mm and an MBC of 15.62 (Table 4; Supplementary Figure S2).

For the G−ve pathogens, *Salmonella typhi* was the most sensitive to EPSR2, with the largest inhibition zone of 31 ± 0.6 mm (Figure 8) and MIC of 15.62 μg/mL. In contrast, *Escherichia coli* was the least susceptible Gram-negative tested, exhibiting the smallest inhibition zone of 17 ± 0.2 and the highest MIC of 250 μg/mL (Supplementary Figure S3). Encouragingly, EPSR2 demonstrated broad-spectrum and potent antibacterial activity against both classes of pathogens, with predominantly bactericidal effects with an overall MBC/MIC ratio ≤ 2 (Table 5).

Following our findings, an EPS extracted from *L. kefiranofaciens* DN1 showed dose-dependent bactericidal and bacteriostatic effects against *S. enteritidis* and *L. monocytogenes* (Jeong et al., 2017). In another study, EPS synthesized by *Lactobacillus* species displayed substantial antibacterial activity, as evidenced by inhibition zones greater than 10 mm in diameter, against *Salmonella enterica* serotype Enteritidis strain ATCC 43972 and the bacterium *Micrococcus luteus* strain Ca6 (Trabelsi et al., 2017). EPS-C70, extracted from *Lactobacillus plantarum* strain C70, resulted in a 2–3 log reduction in viability of *Escherichia coli* and *Staphylococcus aureus* (Ayyash et al., 2020). Another EPS from *Bifidobacterium longum* inhibited cell division processes in several bacterial pathogens, including *Vibrio parahaemolyticus*, *Salmonella Typhimurium*, *Staphylococcus aureus*, and *Bacillus cereus* (Wu et al., 2010). On the other hand, EPSF6 extracted from *Bacillus velezensis* AG6 was tested by MTP assay as an antimicrobial agent, but no considerable results were reported. The authors attributed these negative outcomes to a lack of -S- in the extracted microbial saccharide (Alharbi et al., 2023).

The antibacterial and immune-regulatory activities exhibited by EPSs are closely associated with their physical and chemical characteristics and molecular weight (Ciulla and Gelain, 2023). For example, acidic heteropolysaccharides (HeEPS) containing negatively charged (-PO_4_^3−^) groups were better immune response stimulators than neutral exopolysaccharides without charged functional groups. The negatively charged moieties in HeEPS allowed them to interact with and activate immune cells more strongly (Hidalgo-Cantabrana et al., 2012). Another negatively charged EPS isolated from *Lactococcus lactis* F-mou strain showed more significant inhibition of G+ve bacteria compared to G−ve bacteria, with *Bacillus cereus* ATCC 10702 being the most inhibited G+ve pathogen (Nehal et al., 2019). The researchers hypothesized that the negatively charged EPS resulting from (-SO_4_^2−^) was more effective at interacting with and inhibiting G+ve bacteria. This is because G+ve bacteria’s cell walls possess an overall positive charge, allowing for enhanced electrostatic interactions with the anionic EPS molecules. Therefore, and based on the chemical characterization of the current EPSR2, we may attribute its antibacterial potential to its acidic negative nature due to the presence of galacturonic acid as revealed by HPLC and also due to sulfate residues as evidenced by FT-IR.

Biofilms are communities of bacterial cells embedded in a self-produced polymer matrix attached to a surface. Pathogenic bacteria form biofilms in response to environmental stresses and to evade the host immune system. These biofilms are a significant source of chronic and acute infections because they persist on surfaces and medical devices (Flemming et al., 2016). They also threaten food safety through resistance to standard decontamination. Recent research has focused on controlling, removing, or preventing biofilms due to their impacts on human health (Vestby et al., 2020).

EPSR2 was examined as an anti-biofilm agent. The saccharide exhibited concentration-dependent and broad-spectrum anti-biofilm effects against both tested bacterial spectra, with the highest efficacy at 75% MBC and the Lowest biofilm inhibition seen at 25% MBC across organisms. At 75% of MBC, EPSR2 showed the highest anti-biofilm activity against *B. subtilis, with* 88.3% inhibition (Table 6). In contrast, *S. aureus* displayed the lowest biofilm inhibition of 73.06% at 75% MBC. As the EPSR2 concentration decreased, so did its anti-biofilm potency. At 25% of MBC, the biofilm inhibition dropped to 68.61% for *E. faecalis* and 61.46% for *B. subtilis*. *S. aureus* showed an intermediate susceptibility profile, with 61.42% inhibition at 50% MBC and 38.09% at 25% MBC (Figure 9; Supplementary Table S5).

On the other hand, anti-biofilm activity against G−ve pathogens: At 75% of MBC, EPSR2 displayed the highest biofilm inhibition of 96.63% against *E. coli*, followed by 86.91% against *S. typhi*, 86.45% for *P. aeruginosa*, and 84.36% against *K. pneumoniae* (Figure 10). Even at 25% MBC, EPSR2 reduced biofilm formation by 84.13% in *E. coli*, 58.99% in *K. pneumoniae*, 54.67% in *S. typhi*, and 46.17% in *P. aeruginosa*. *E. coli* was most susceptible (Table 7; Supplementary Table S6), while *P. aeruginosa* was relatively resistant. EPSR2 displayed a broad spectrum, concentration-dependent anti-biofilm activity against both G+ve and G−ve bacteria, although its potency varies across bacterial strains.

Accordingly, Rani et al. reported that the EPS extracted from *L. gasseri* FR4 exhibited the highest antibiofilm inhibition 56% against *L. monocytogenes* MTCC 657 (56% inhibition) and the lowest inhibition 19.2% against *Enterococcus faecalis* (Rani et al., 2018). Additionally, EPS from *L. plantarum* WLPL04 inhibited *P. aeruginosa* and *E. coli* O157:H7 biofilm formation by 47.02 and 25.82%, respectively (Liu et al., 2017). Another EPS extracted from *L. plantarum* YW32 suppressed G+ve and G−ve biofilm formation, as reported by Wang et al. (2015). The author proposed that EPS may disrupt biofilm activity by altering bacterial cell surfaces, preventing initial adherence of bacterial cells to surfaces, or down-regulating gene expression involved in biofilm formation through signaling effects (Wang et al., 2015). The EPS synthesized by *L. coryniformis* NA-3 exhibited the strongest disruptive effect on pre-formed biofilms of *S. typhimurium* and *B. cereus* compared to other strains tested. The EPS disrupted 80% of *S. typhimurium* and 90% of *B. cereus* pre-formed biofilms (Xu et al., 2020). On the other hand, EPSF6 isolated from *B. velezensis* AG6 showed no biofilm activity towards *E. coli* and *S. aureus* by MTP assay (Alharbi et al., 2023).

One hypothesized approach to hinder or eradicate biofilms is blocking quorum sensing (QS) systems, which disrupts early adherence, autoaggregation, and consequent mature biofilm formation. Quorum sensing regulates key behaviours like motility, virulence, and antibiotic production (Preda and Săndulescu, 2019; Rather et al., 2021). Quorum sensing relies on autoinducers and chemical signaling molecules that bacteria produce, release, and detect. Bacterial pathogens recognize EPSs as foreign molecules. Due to their size and charge, EPS cannot enter pathogen cells to act internally. Thus, EPS may interfere with biofilm signaling molecules or block glycocalyx pathogen surface receptors, causing quorum quenching and impeding biofilm formation (Algburi et al., 2017; da Silva et al., 2021).

Different EPS forms from diverse sources exhibit varying degrees of activity, which are determined, as mentioned earlier, by their chemical structure, functional group residuals, side chains, degree of branching, and chemical substituents (Mishra and Jha, 2013). Consequently, Microbial EPSs have been modified to enhance their bioactivity through physical, chemical, and biological processes. Due to the diverse biological actions of numerous naturally occurring sulfated polysaccharides, sulfation is one successful modification employed besides acetylation and phosphorylation. A new approach to modifying EPSs involves using mutational methods to alter the nucleotide sequences of microbes through biomolecular modification. UV radiation is an easy, low-cost way to generate mutations. Mutant bacterial strains can produce EPSs with different properties and bioactivities (More et al., 2014; Poli et al., 2017).

Molecular techniques help confirm the pathways involved in polysaccharides that stimulate immunity. A better understanding of these mechanisms behind EPSs will promote the development and modification of bioactive EPS for other biotechnological and nano applications. Also, improving fermentation strategies can increase the yield and productivity of EPSs from microbes. New or enhanced fermentation techniques that are cost-effective and robust are important and highly required for optimizing microbial EPS production on the industrial scale (Mahmoud et al., 2021; Eswar et al., 2023).

## 5 Conclusion

This study isolated a novel exopolysaccharide (EPSR2) from the marine bacterium *Bacillus licheniformis* strain LHG166 obtained from the Red Sea. Structural characterization revealed EPSR2 to be a sulfated, acidic heteropolysaccharide composed of five different monosaccharides. Comprehensive biological screening uncovered diverse bioactivities, including appreciable antioxidants. More significantly, EPSR2 exhibited potent and selective antifungal effects against the pathogenic yeast *Candida albicans*. Broad-spectrum antibacterial screening also showed efficacy against both G+ve and G−ve bacterial pathogens, with the highest potency seen against *Enterococcus faecalis* and *Salmonella typhi*. At sub-MICs, EPSR2 displayed concentration-dependent anti-biofilm effects against clinically relevant bacteria. Such results indicate the promise of EPSR2 as a multifaceted therapeutic lead compound, possessing antioxidant, antifungal, antibacterial, and anti-biofilm properties. The potent inhibitory activity against specific pathogens reveals potential for targeted treatment of candidiasis, enterococcal infections, salmonellosis, and device-related biofilm infections.

Further development is warranted, including toxicity, *in vivo* verification studies, gut microbiome interactions, and pharmacological optimization. Overall, this work adds to the growing knowledge of marine bacteria as prolific producers of bioactive natural products like EPSR2 from marine *Bacillus licheniformis* LHG166 for drug discovery efforts. Bioprospecting marine microbes can provide new weapons to fight resistant infections and oxidative stress, addressing pressing healthcare challenges.

## Data availability statement

The original contributions presented in the study are included in the article/Supplementary material, further inquiries can be directed to the corresponding author.

## Author contributions

NKA: conceptualization, methodology, writing – original draft, writing – review & editing. ZFA: methodology, investigation, formal analysis, validation. HMA: data curation, visualization, formal analysis, writing – review & editing. AMAS: methodology, software, data curation, writing – review & editing. MOIA: methodology, investigation, writing – original draft, writing – review & editing. MGE: visualization, methodology, data curation, software. GSA: visualization, data curation, methodology, software. ME: Formal analysis, investigation and methodology. EMK: methodology, investigation and writing – review & editing. AG: Conceptualization, methodology, writing – review & editing, supervision, writing – original draft and supervision.
