# Supplementary material for: Tapping the biosynthetic potential of marine Bacillus licheniformis LHG166, a prolific sulphated exopolysaccharide producer: structural insights, bio-prospecting its antioxidant, antifungal, antibacterial and anti-biofilm potency as a novel anti-infective lead
Source: Front Microbiol. 2024 Apr 10;15:1385493. doi: 10.3389/fmicb.2024.1385493 (PMC11039919; doi:10.3389/fmicb.2024.1385493)

**Supplementary Data**

**Tapping The Biosynthetic Potential of Marine *Bacillus* *Licheniformis* LHG166, A Prolific Sulphated Exopolysaccharide Producer: Structural Insights, Bio-Prospecting Its Antioxidant, Antifungal, Antibacterial And Anti-Biofilm Potency As A Novel Anti-Infective Lead**

| **Culture and Morphological Features** | ***Bacillus licheniformis LHG166*** |
| --- | --- |
| **Gram stain** | Gram +ve  (short rod) |
| **Colony surface** | smooth |
| **Colony texture** | rough |
| **Color** | Pale yellow |
| **Elevation** | Flat |
| **Edge** | entire |
| **Whole colony** | Irregular  Large colony |
| **Pigmentation** | No |
| **Opacity of the bacterial colony** | Opaque |
| **Anaerobic condition** | + |

**Table S1.** Culture and Morphological Characteristics of *Bacillus licheniformis* LHG166

**Table S2.** Physiological and Biochemical Characteristics of *Bacillus licheniformis* LHG166

| ***Bacillus licheniformis LHG166*** | **Physiological and Biochemical Features** |
| --- | --- |
| + | **Starch hydrolysis** |
| + | **Catalase test** |
| + | **urease** |
| + | **Voges- Proskauer test** |
| + | **Simmon citrate test** |
| + | **Nitrate reduction** |
| **Carbohydrates fermentation** | |
| + | **Glucose** |
| + | **Maltose** |
| + | **Sucrose** |
| + | **Lactose** |
| + | **Arabinose** |
| + | **Mannitol** |
| + | **Starch** |

| **TAC ascorbic con. µg/ml** | **Absorbance** | **FRAP ascorbic con. µg/ml** | **Absorbance** |
| --- | --- | --- | --- |
| 1000 | 1.295 | 1000 | 1.566 |
| 800 | 0.963 | 800 | 1.236 |
| 600 | 0.692 | 600 | 0.997 |
| 400 | 0.455 | 400 | 0.756 |
| 200 | 0.211 | 200 | 0.433 |
| 100 | 0.124 | 100 | 0.317 |

**Table S3.** Ascorbic acid concentrations and absorbance in TAC and FRAP antioxidant assays

**Table S4.** EPSR2 antioxidant values in TAC and FRAP assays

| **EPSR2** | **TAC (equivalent (AAE) µg/mg)** |  |  | **Mean** | **STD** | **SE** |
| --- | --- | --- | --- | --- | --- | --- |
|  | 106.1 | 106.28 | 105.84 | 106.07 | 0.221 | 0.072 |
|  | Absorbance * | | | | | |
|  | 0.646 | 0.648 | 0.645 |  |  |  |
| **EPSR2** | **FRAP (equivalent (AAE) µg/mg)** |  |  | **Mean** | **STD** | **SE** |
|  | 59.3 | 60.8 | 60.2 | 60.1 | 0.8 | 0.246 |
|  | Absorbance ** | | | | | |
|  | 0.769 | 0.784 | 0.778 |  |  |  |

* To ensure that the sample concentration fell within the linear range, it was analyzed at 5.0 mg/mL, and the result was divided by five

** To fall within the linear range, the 10 mg/mL sample was analyzed, and the result divided by ten

**
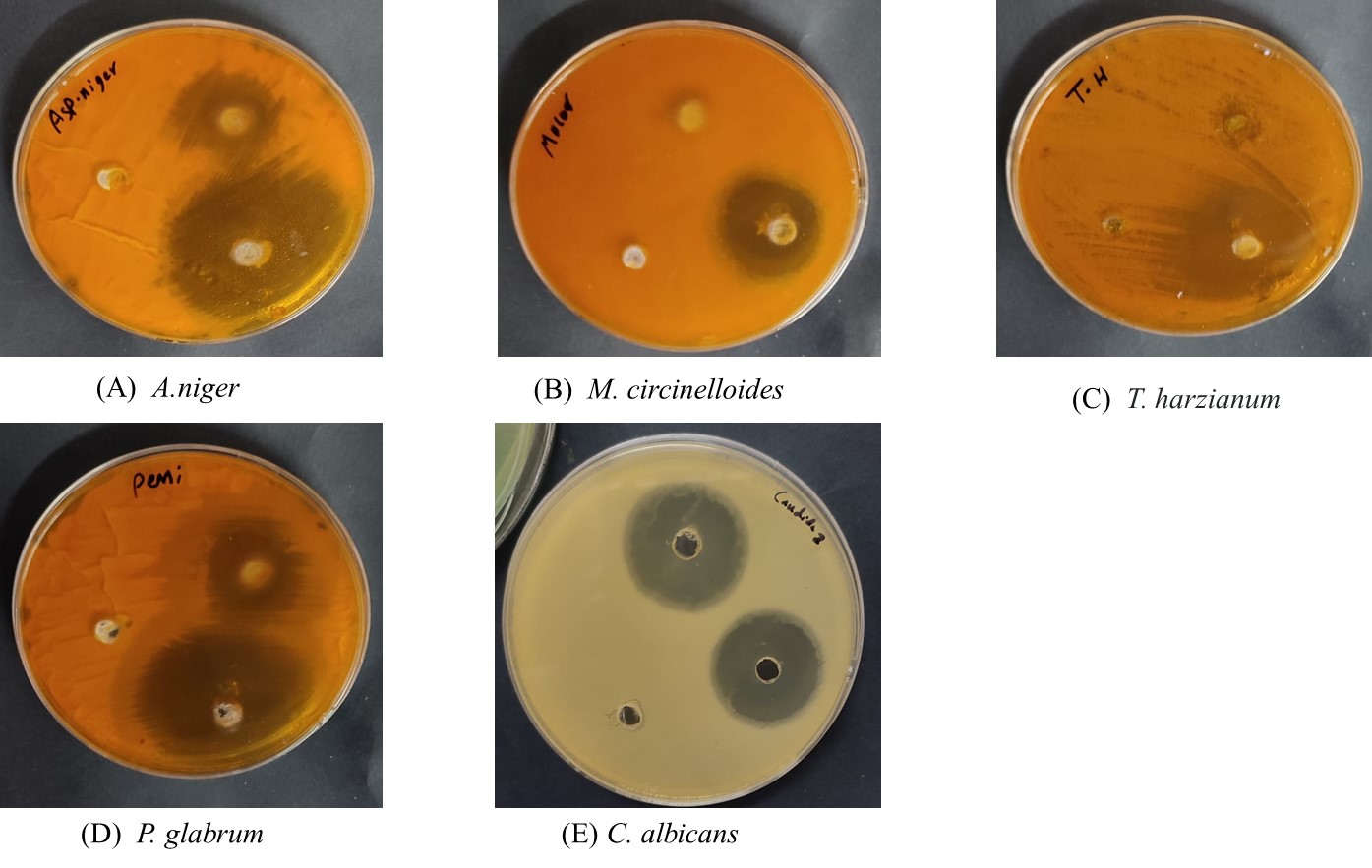
**

**Figure S1.** EPSR2 and Fluconazole antifungal activity represented as inhibition zone (mm) against filamentous fungi.


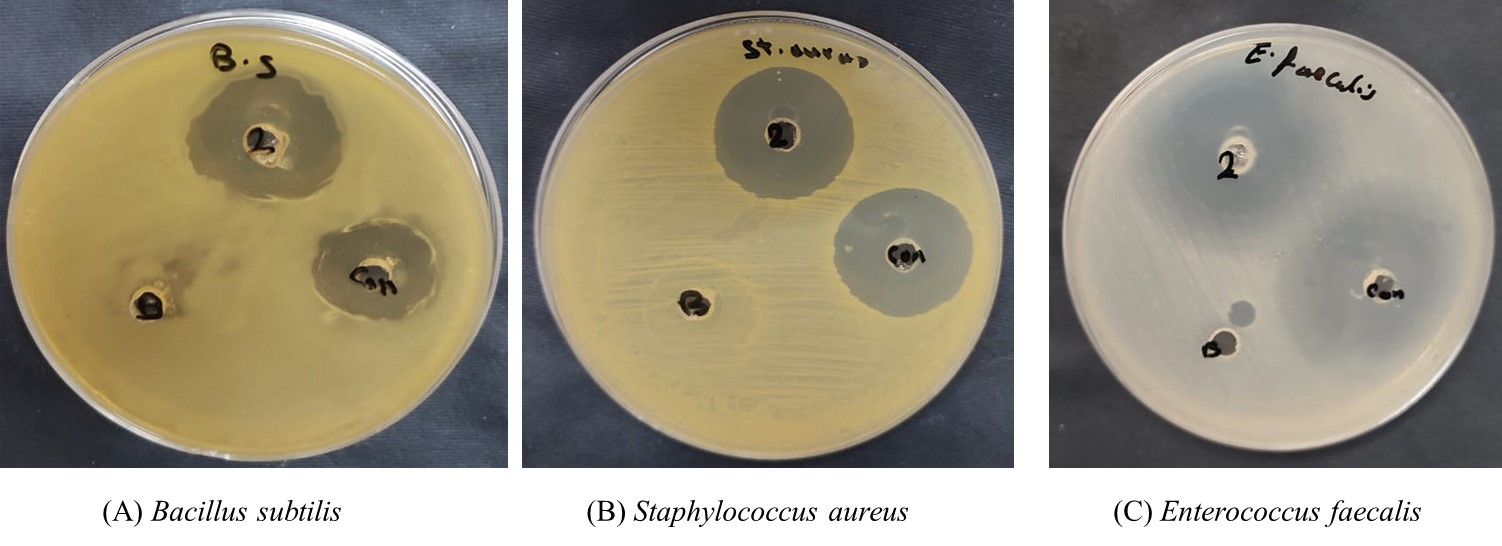


**Figure S2.** Inhibition Zones of EPSR2 and Gentamicin against G+ve bacteria

**
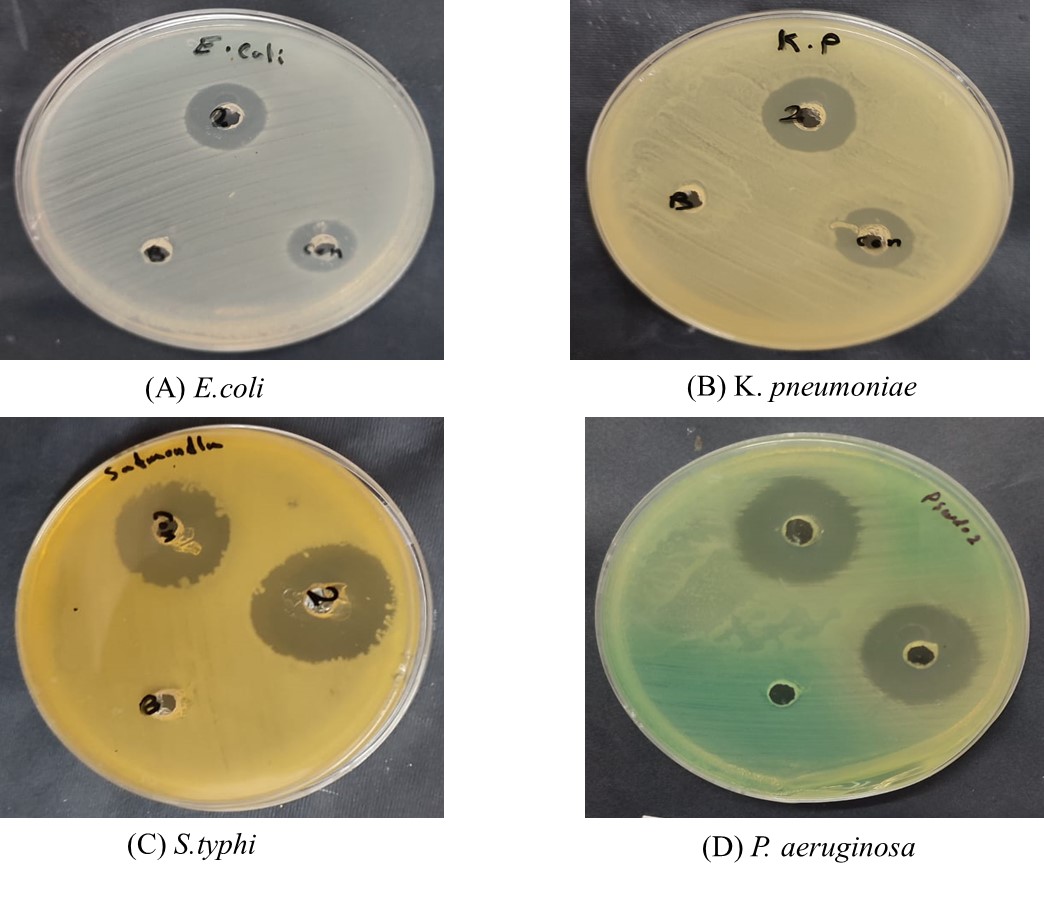
**

**Figure S3*.*** Inhibition Zones of EPSR2 and Gentamicin against G-ve bacteria

| **EPSR2 - MBC% of *E. faecalis* Anti-biofilm %** |
| --- |
| Blank (Media only) - |
| Media+Organism (Cont.) - |
| 25% of MBC 68.61 |
| 50% of MBC 81.10 |
| 75% of MBC 87.93 |
| **EPSR2 - MBC% of** ***S. aureus* Anti-biofilm %** |
| Blank (Media only) - |
| Media+Organism (Cont.) - |
| 25 % of MBC 38.09 |
| 50% of MBC 61.42 |
| 75% of MBC 73.06 |

**Table S5.** EPSR2 Antibiofilm activity against E. faecalis and S. aureus at 25, 50, and 75% MBC.


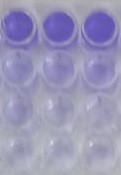


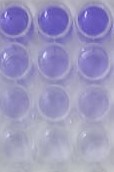


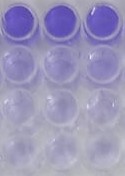

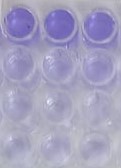
 **Table S6.** EPSR2 Antibiofilm activity against K. pneumoniae, S.typhi, and P. aeruginosa at 25, 50, and 75% MBC.

| **EPSR2 - MBC% of** ***K. pneumoniae*  Anti-biofilm %** |
| --- |
| Blank (Media only) - |
| Media+Organism (Cont.) - |
| 25% of MBC 58.99 |
| 50% of MBC 78.36 |
| 75% of MBC 84.36 |
| **EPSR9 - MBC% *S.typhi* Anti-biofilm %** |
| Blank (Media only) - |
| Media+Organism (Cont.) - |
| 25 % of MBC 54.67 |
| 50% of MBC 67.07 |
| 75% of MBC 86.91 |
| **EPSR2 - MBC% *P. aeruginosa* Anti-biofilm %** |
| Blank (Media only) - |
| Media+Organism (Cont.) - |
| 25 % of MBC 46.17 |
| 50% of MBC 65.13 |
| 75% of MBC 86.45 |


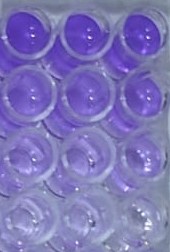

Supplement: Supplementary file 1 [file Table_1.DOCX]
